# The Effect and Safety of Rapid and Gradual Urinary Decompression in Urine Retention: A Systematic Review and Meta-Analysis

**DOI:** 10.3390/medicina58101441

**Published:** 2022-10-13

**Authors:** Meng-Yu Wu, Jer-Ruey Chang, Yi-Kung Lee, Po-Chen Lin, Tou-Yuan Tsai

**Affiliations:** 1 Department of Emergency Medicine, Taipei Tzu Chi Hospital, Buddhist Tzu Chi Medical Foundation, New Taipei City 231, Taiwan; skyshangrila@gmail.com (M.-Y.W.); avenlin6059@gmail.com (P.-C.L.); 2 School of Medicine, Tzu Chi University, Hualien 970, Taiwan; lyg1968@gmail.com; 3 Emergency Department, Dalin Tzu Chi Hospital, Buddhist Tzu Chi Medical Foundation, Chiayi, 622, Taiwan; imgr1985@gmail.com

**Keywords:** acute urinary retention, rapid decompression, hematuria, circulatory collapse

## Abstract

*Background and objectives:* Trials to evaluate the effect and safety of rapid and gradual urinary decompression have been published for decades. Due to inconclusive results, this study aimed to assess whether rapid bladder decompression increased complications in adults with acute urinary retention. *Materials and Methods:* We searched the *Cochrane Library*, *EMBASE*, *Google Scholar,* and *PubMed* databases for articles published from the database inception to 31 August 2021. Studies that compared the effects and complication rates of rapid and gradual urinary decompression in adults with acute urinary retention were included. The primary outcome was post-decompression hematuria, while the secondary outcome was circulatory collapse. Meta-analyses were conducted using random effects models. Sensitivity analyses, tests for publication bias, and trial sequential analyses were conducted. The PROSPERO registration number is CRD42021233457. *Results:* Overall, four articles were included in the comprehensive analysis, and 435 participants met all the eligibility criteria. In the primary meta-analysis of all four study groups, rapid urinary decompression did not increase the risk of post-decompression hematuria (RR = 0.91; 95% CI: 0.62 to 1.35; *p* = 0.642). The *I*^2^ statistic was 0.0% (*p* = 0.732), indicating no substantial heterogeneity. In the meta-analysis of randomized controlled studies, the result did not change (RR = 0.89; 95% CI: 0.31 to 2.52; *p* = 0.824). The Egger’s test and Begg test (*p* = 0.339 and 0.497, respectively) indicated the absence of statistical evidence of publication bias. Leave-one-out sensitivity analysis was conducted and showed the pooled results were robust. In secondary outcome, there were no reported events of circulatory collapse in the current studies. *Conclusions:* The currently available data suggest that rapid urinary decompression is an effective and safe method with a complication rate similar to that of gradual decompression in an acute urinary retention population. Further large-scale randomized studies are required.

## 1. Introduction

Urinary retention (UR) is a urological emergency that commonly occurs in men aged >60 years and increases with age [1,2]. The most common underlying mechanisms are outflow obstruction, neurologic defects, or weak detrusor muscle [3,4]. Acute urinary retention (AUR) generally presents with lower abdominal pain and an inability to pass urine. In the elderly population, particularly those with dementia and cognitive impairment, AUR may present with non-specific symptoms, such as acute altered mental status or sepsis. Acute-on-chronic UR may often go unnoticed and progress over time with growing residual volumes, which usually present with painless overflow incontinence. Unspecific symptoms of UR may delay the diagnosis and lead to hydronephrosis. The initial management of UR includes early drainage by transurethral or suprapubic catheterization. Early drainage reduces the risk of urinary tract infections and renal failure. The two major management strategies promoted are rapid decompression (RD) and gradual decompression (GD).

Rapid complete bladder decompression immediately reduces painful sensations and increases the rate of potential complications, including transient hematuria, circulatory collapse, and obstructive diuresis. To avoid potential complications, the conceptual treatment of UR in clinical practice is GD. However, the evidence is generally weak [5]. Moreover, GD is complex and time-consuming. Few studies have focused on the release rates and complication risks associated with RD and GD. In a review by Nyman et al. [6], the complication rate was reported to be low in RD, and they concluded that RD was safe. Nevertheless, more studies have recommended GD to avoid complications [7]. To date, debate persists over which therapeutic strategy is better for UR.

Our study aimed to provide strong evidence to confirm the effects of RD and GD and to focus on the complications of RD and GD in UR by conducting a meta-analysis and systematic review of the relevant literature.

## 2. Methods

### 2.1. Protocol

This systematic review was conducted and reported in accordance with the Preferred Reporting Items for Systematic Reviews and Meta-Analyses Statement (The PRISMA 2020 statement) [8]. The Institutional Review Board of Dalin Tzu Chi Hospital, Buddhist Tzu Chi Medical Foundation, Taiwan, approved the protocol (No. B11001007). The PROSPERO registration number is CRD42021233457.

### 2.2. Databases and Search Strategy

We searched the *Cochrane Library*, *EMBASE*, *Google Scholar*, and *PubMed* databases for articles published from the database inception to 31 August 2021. No limits were applied to our Boolean search strategy, which included keywords “urine retention”, “intravesical pressure”, “decompression”, “drainage”, “hematuria”, “hypotension”, and “diuresis”. References from the retrieved articles were also examined to identify other relevant articles. The details of the search strategy are provided in Table 1.

Studies were included in the systematic review if (1) the participants were diagnosed with UR; (2) release rates of different urinary bladder decompression methods were compared; (3) the outcome measurements included the number of hematuria events, blood pressure changes, or diuresis; and (4) studies were conducted in humans. Studies were excluded if they were irrelevant to the study’s aim, were conducted in animals, lacked a placebo group, or were published as review articles, case reports, editorials, or letters.

### 2.3. Data Extraction and Assessment of Methodological Quality

Two reviewers (M.Y.W. and J.R.C.) independently screened the titles and abstracts of all articles identified by the search strategy. Inter-reviewer disagreements concerning the inclusion or exclusion of a study were resolved by consensus and, if necessary, consultation with a third reviewer (T.Y.T.).

The Cochrane Collaboration’s tool was used to assess the risk of selection, performance, detection, attrition, and reporting biases in the included randomized trials and nonrandomized trials. We used the revised risk of bias (RoB 2) assessment for randomized controlled studies and the “Risk Of Bias In Non-randomized Studies of Interventions” (ROBINS-I) assessment for non-randomized studies. [9,10] All co-authors discussed and made final decisions about the overall risk of bias in the included trials. If data were not readily available or clear, we contacted the first authors and corresponding authors to obtain further information. If studies were found to be at a high risk of bias, meta-analyses stratified by study quality were performed. Both reviewers independently extracted data from the articles selected for inclusion. The extracted data included the name of the first author, year of publication, number of participants, sex, urinary catheter size, method of catheterization, number of individuals with hematuria, blood pressure changes, and diuresis.

### 2.4. Data Collection, Data Processing, and Primary Data Analysis

The pooled relative risk (RR) with corresponding 95% confidence intervals (CIs) for each outcome of interest were calculated. The main outcome measure was the RR of hematuria in participants with urinary bladder decompression. Random effects models were selected for these analyses. Between-study heterogeneity was evaluated using *I*^2^ statistics. If more than 10 studies were included in each outcome of the meta-analysis, we used a funnel plot to assess publication bias or small-study bias. The Egger regression asymmetry test and Begg adjusted rank correlation test were applied to assess potential publication bias [11,12]. We also conducted sensitivity analysis to evaluate the influence of each study on the overall pooled estimate. For the zero cells dealing, we added 0.5 to all cells of the 2 × 2 table for the study. All analyses were conducted using STATA version 15.1 (StataCorp, College Station, TX, USA). All statistical tests were two-sided and considered significant when the *p*-values were ≤0.05. Trial sequential analysis (TSA) was also conducted using TSA software (version 0.9.5.10 Beta, Copenhagen Trial Unit, Copenhagen, Denmark) to evaluate whether the results of the updated meta-analysis were conclusive, with an alpha of 5%, a power of 80%, and an RR reduction of 20% given the incidence rate in the control group.

### 2.5. Grading of the Certainty of Evidence

M.Y.W. and J.R.C. assessed the certainty of evidence (CoE) using the “Grading of Recommendations Assessment, Development and Evaluation” (GRADE) methodology to assess the quality of evidence with individual endpoints. After step-by-step evaluation, CoE was classified as high, moderate, low, or very low [13]. 

## 3. Results

### 3.1. Search Results

**Figure 1:**
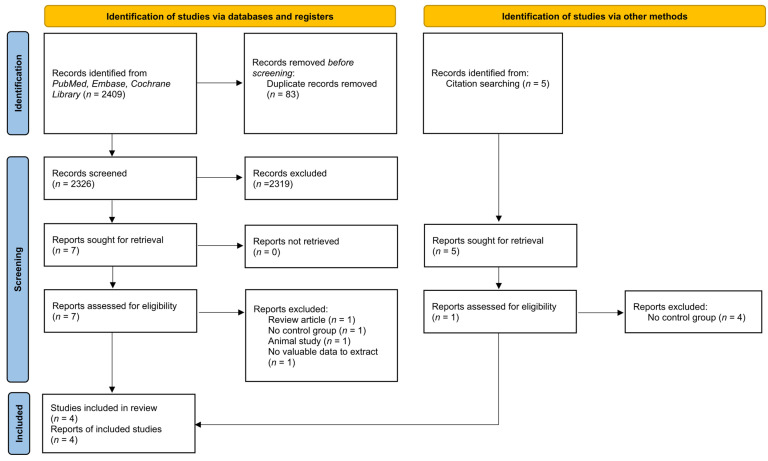
PRISMA (Preferred Reporting Items for Systematic Review and Meta-analysis) 2020 flow diagram for new systematic reviews which included searches of databases and other sources.

The literature search and study selection processes are summarized in Figure 1. Five publications were retrieved to be included for analyses by a manual search of the references [14,15,16,17,18]. After the exclusion of duplicate, non-relevant, and other studies that met exclusion criteria based on a screening of article titles and abstracts, 12 potentially relevant studies were retrieved for the full review. Twelve potential studies were included. Five studies reported hematuria or circulatory collapse events after bladder decompression without a comparison of rates of different methods [15,16,17,18,19]. As a result, four published articles met all eligibility criteria after a careful review [14,20,21,22].

A total of four study groups with 435 participants were enrolled. In four included studies, there were two studies that included acute urinary retention: one included chronic urinary retention and one study included both acute and chronic urinary retention population. The characteristics of these studies and the participants are listed in Table 2. Two studies were randomized trials [20,22]. All studies included a small number of subjects except the randomized control trial conducted by Boettcher and colleagues in 2013 [20]. Of note, all studies included male participants. The methodological quality of the two randomized controlled studies and two non-randomized studies was appraised using RoB 2 and the ROBINS-I, respectively, as depicted in Table 3 and Table 4.

### 3.2. Main Outcomes

**Figure 2:**
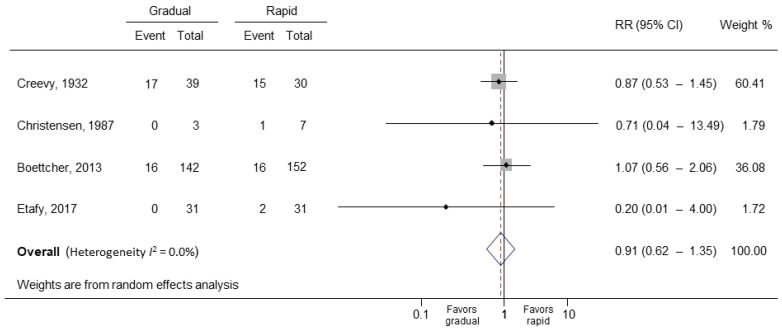
Forest plot of hematuria in gradual and rapid decompression groups. RR, relative risk; CI, confidence interval.

**Figure 3:**
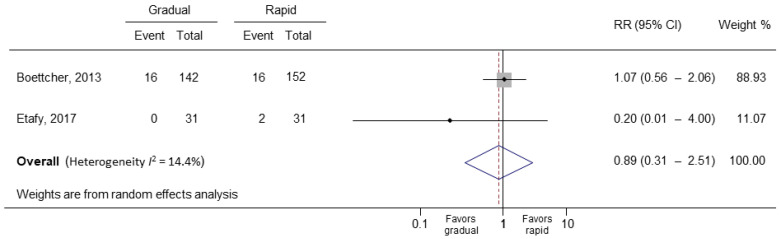
Forest plot of randomized controlled studies. RR, relative risk; CI, confidence interval.

**Figure 4:**
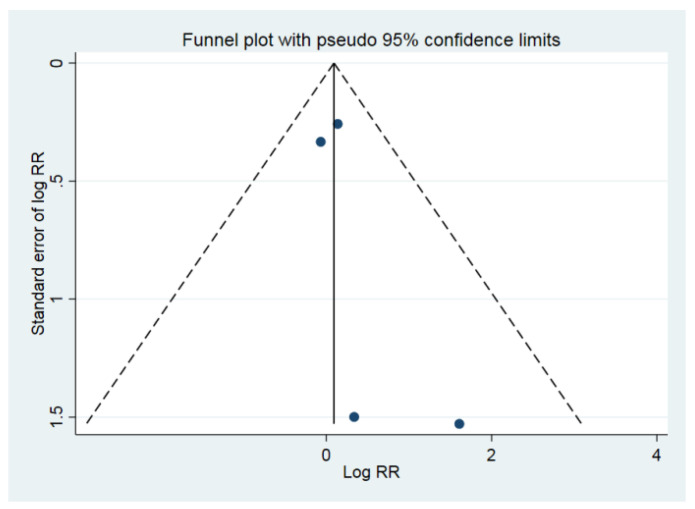
The funnel plot of included studies for publication bias. RR, relative risk.

**Figure 5:**
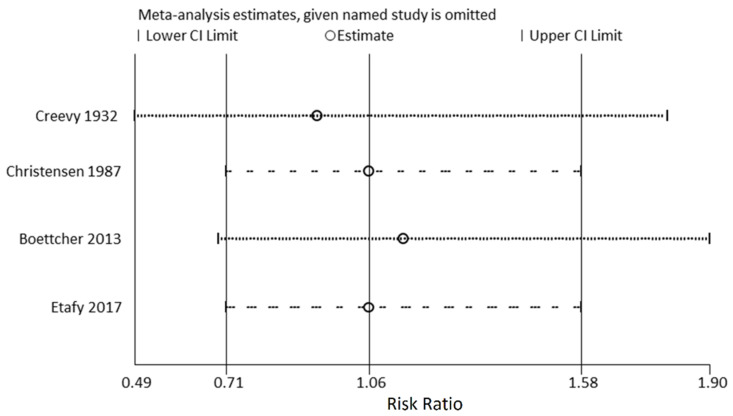
Sensitivity analysis for hematuria. CI, confidence interval.

In the primary meta-analysis of all four study groups, rapid bladder decompression in UR did not increase the risk of hematuria (RR = 0.91; 95% CI: 0.62 to 1.35; *p* = 0.642) (Figure 2) [14,20,21,22]. The *I*^2^ statistic was 0.0% (*p* = 0.732), indicating low substantial heterogeneity. After including randomized controlled studies only, the *I*^2^ statistic became 14.4% (*p* = 0.28), and the result did not change (RR = 0.89; 95% CI: 0.31 to 2.52; *p* = 0.824) (Figure 3) [20,22]. The Egger’s test and Begg test (*p*-values, 0.339 and 0.497, respectively) indicated the absence of statistical evidence of publication bias after excluding our presumed high-risk bias articles. The funnel plot of included studies for publication bias is shown in Figure 4. Sensitivity analysis was conducted by removing one trial at a time to determine the influence of each study on the pooled analysis. The pooled results were robust. For example, removing the study conducted by Creevy conducted in 1932 [14] only changed the pooled estimate from 0.91 to 0.93 (95% CI 0.49–1.80; *p* = 0.540; Figure 5).

**Figure 6:**
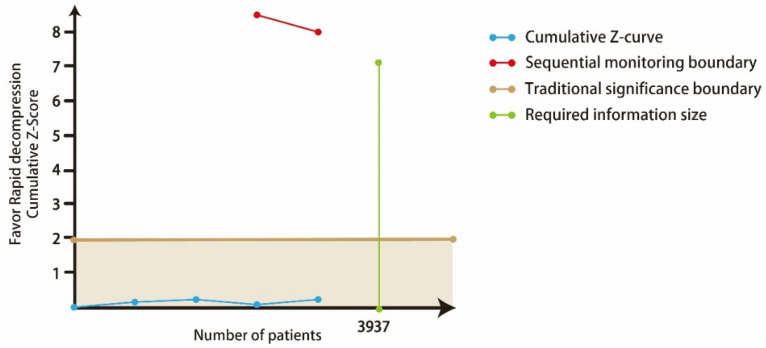
Trial sequential analysis for hematuria.

However, in the TSA analysis of hematuria, although the cumulative Z-curve did not pass the traditional significance boundary, it did not reach the required information size, indicating a false-negative result (Figure 6). In addition, this meta-analysis is solely based on unadjusted observational study results and should be interpreted carefully. Three studies reported events of hematuria after RD without comparison, and the details are presented in Table 5 [15,17,19].

Regarding secondary outcomes, seven studies reported changes in blood pressure after decompression [14,16,18,20,21,22]. Four of the studies reported comparisons between RD and GD [14,20,21,22]. Of note, no events of significant hypotension were reported in all studies.

### 3.3. CoE Score

The CoE is presented in Table 6. Regarding study limitations, we identified very serious risk of bias regarding two studies with some concern and one study with serious bias for overall RoB being enrolled in both endpoints. The small number of cases limited not only the assessment of hematuria events in different decompression methods but also the precision of every outcome measurement. According to the results of the TSA, the sample size for risk of hematuria in RD was not large enough. Overall, the CoE showed very low risk of hematuria.

## 4. Discussion

To our knowledge, this is the first meta-analysis to evaluate the relationship between different decompression rates and complications in patients with acute urinary tension. In pooled analyses, we observed that the RD method for AUR did not increase the risk of hematuria (RR = 0.91; 95% CI 0.62–1.35; *p* = 0.642; *I*^2^ = 0.0%). RD did not increase circulatory collapse events compared to the GD group. Our results provide evidence that RD is effective and safe for patients with AUR.

Hematuria and hypotension have been a concern as complications of the rapid release of urinary tract obstruction, causing injury and resulting in hemorrhage. Based on this concept, GD was recommended in previous literature. However, another hypothesis has been promoted that hematuria results from tissue damage with edema and hemorrhage due to raised intravesical pressure. After rapid decompression, the sudden release of compressed vessels with renal and bladder may cause bleeding [23,24,25]. In vitro, bladder function was impaired after urinary retention. Overstretching leaded to diffuse/focal submucosal hemorrhages. A diffused edema in areas of outer mesoaxons and cytoplasmic processes of Schwann cells, accompanied by a rupture of the surrounding basement membrane, was shown in electron microscopic changes [26,27]. This results concluded that hematuria after treatment of urinary retention is contributed to tissue damage rather than the mode of decompression. In addition, the other etiologic factors, including infection, malignancy, and iatrogenic trauma, may also result in hematuria from GD and RD. Our results were compatible with previous evidence of RCT and animal studies: we found no significant difference of risk for hematuria between RD and GD method.

Hematuria may occur in 2–16% of patients with UR after RD [6]. Rapid and complete bladder emptying may cause injury to the urinary tract, leading to hemorrhage. A typical case was reported by Gabriel and Suchard in which gross hematuria occurred after three hours of rapid drainage of over two liters of urine [28]. Post-decompression hematuria generally resolves with irrigation; it may prolong retention time in the emergency department and increase the risk of urinary catheter replacement. In previous recommendations, GD of an obstructed bladder was considered more suitable than RD to avoid complications such as hematuria, hypotension, and post-obstructive diuresis although this concept is debatable. In our results, the risk ratio of post-decompression hematuria between the two groups was not significant. Another concern is that the risk and severity of post-decompression hematuria may increase and worsen in high-risk populations who receive antiplatelet or anticoagulation treatments. In a study by Boettcher et al. [20], which was a randomized controlled trial of 294 patients with UR, no statistically significant differences between the rapid and gradual groups were observed. In the subgroup analysis of anticoagulation treatment, such as warfarin, clopidogrel, aspirin, and heparin, there was no significant difference (*p* = 0.605). Interestingly, the severe events of hematuria in the GD group (37.4%) seemed to be more severe than in the RD group (25%). In our analyses, the study presented evidence that GD is not superior to RD in post-decompression hematuria.

Post-decompression circulatory collapse, including transient hypotension, has been reported [29]. AUR and pain sensation leads to high blood pressure due to a higher sympathetic tone and triggering urinary vesicovascular reflex [16,18]. Acute release of bladder wall tension reflex causes vasodilatation with post-decompression transient hypotension. In the healthy population, post-decompression transient hypotension may not cause serious clinical consequences due to dynamic changes in blood pressure within the normal blood pressure range [18]. In our subgroup analysis, there were no events of post-decompression circulatory collapse reported in the seven included studies. Although a previous review article mentioned this concern, our data did not support that GD for AUR is more effective in preventing circulatory collapse than RD. Our result was similar to that of Boettcher et al. [20], who reported that no circulatory collapse event after RD or GD was found. In both groups, blood pressure decreased after catheterization, but there was no significant difference in both groups before and after catheterization [20]. Likewise, initial tachycardia decreased after catheterization, but there was no significant difference in both groups before and after catheterization [20].

Our study has several limitations. First, few comprehensive studies investigated this issue, and only two randomized clinical trials reported interesting outcomes. Half of the included studies were retrospective studies. All studies had a small number of subjects except the study by Boettcher et al. [20], which was solely based on unadjusted observational study results. A great heterogeneity regarding the sample size was noted in our included studies. In addition, the population in included studies involved acute and chronic urinary retention patients, which are different mechanisms causing urinary retention and also results in different clinical outcomes after RD or GD. Therefore, the strength of evidence for the study should be interpreted carefully. Nevertheless, the issue is definitely of clinical importance, and observational studies are currently available forms of evidence. Second, no event of post-decompression circulation collapse was reported, which may have resulted from a lower incidence rate and small sample size even in the largest randomized clinical trial by Boettcher et al. [20]. Finally, although all included studies provided consistent results and low heterogeneity in our meta-analysis (*I*^2^ = 0%), the current results do not pass the traditional significance boundary in TSA analysis. Although few clinicians focused on the hematuria and hypotension after RD and GD due to low incidence of hypotension and self-limited hematuria, GD is more complex and time-consuming than RD. In the future, larger randomized clinical trials with other clinical outcomes, such as rate of urinary tract injection and cost–benefit outcome, are necessary and warranted to obtain definitive results.

## 5. Conclusions

Compared to GD, patients with AUR receiving rapid complete decompression did not have a higher risk of hematuria or circulatory collapse. Further large-scale randomized control studies are warranted.

## Figures and Tables

**Table 1:** Search strategy examples.

*PubMed*	(“Urinary Retention” [All fields] OR “Bladder” [All fields] OR “Intravesical pressure” [All fields]) AND (Decompression* [All fields] OR Decompress* [all fields] OR drainage [all fields] OR Rapid [All fields] OR Gradual [All fields] OR Rate [All fields] OR “Urinary Catheterization” [All fields] OR “Urinary Catheters” [All fields]) AND (Hematuria [All fields] OR hypotension [All fields] OR diuresis [All fields])
*Embase*	(“Urinary Retention” OR “Bladder” OR “Intravesical pressure”) AND (Decompression OR Decompress OR drainage OR “Urinary Catheterization” OR “Urinary Catheters”) AND (Hematuria OR bleeding OR hypotension OR Circulation OR diuresis)

**Table 2:** Characteristics of included studies.

Study	Year	Mean Age (Year)	Male (%)	Study Size	Acute or Chronic Urinary Retention	Catheter Size	Method of Decompression	
							Rapid	Gradual
Creevy	1932	67.2	100 *	69	Chronic	NA	Emptied promptly at a single session	Decompressed gradually in from one to five days
Christensen	1987	74.5 ^†^	100	10	Acute	PVC Foley 3-way catheter 20 Fr.	Complete continuous drainage	Fractionated drainage was performed in decrements of 100 cm^3^, allowing a steady state to elaborate between drainage periods
Boettcher	2013	72.5	100	294	Both	Chosen with regard to patient history	Drained completely by placing the drainage bag at a lower level than the bladder.	After each 200 mL of urine drained, the catheter was clamped for 5 min and then reopened until the bladder was completely empty
Etafy	2017	63.8	100 *	62	Acute	Foley catheter, no mentioned size	Managed by rapid drainage of the bladder	The first 100 mL were immediately evacuated, then the rest was evacuated gradually in 2 h

* The participants were all diagnosed with benign prostate hyperplasia; ^†^ The age reported as median.; NA: not available.

**Table 3:** Risk of bias for randomized controlled studies (RoB 2).

Risk of Bias Domain	Boettcher, 2013	Etafy, 2017
Randomization process	Low risk	Some concerns
Deviations from intended interventions	Some concerns	Low risk
Missing outcome data	Low risk	Low risk
Measurement of the outcome	Low risk	Low risk
Selection of the reported result	Low risk	Some concerns
**Overall risk of bias**	**Some concerns**	**Some concerns**

**Table 4:** Risk of bias in non-randomized studies (ROBINS-I).

Risk of Bias Domain	Creevy, 1932	Christensen, 1987
Bias due to confounding	Serious	Moderate
Bias in selection of participants into the study	Moderate	Low
Bias in classification of interventions	Serious	Low
Bias due to deviations from intended interventions	Serious	Moderate
Bias due to missing data	Serious	Low
Bias in measurement of outcomes	Moderate	Moderate
Bias in selection of the reported result	Moderate	Low
**Overall bias**	**Serious**	**Moderate**

**Table 5:** Events of hematuria and circulatory collapse in rapid and gradual bladder decompression groups.

Study	Year	Mean Age (Year)	Male (%)	Study Size	Events of Complication in Different Rate of Bladder Decompression *n* (%)	
					Gradual	Rapid
Hematuria						
Creevy	1932	67.2	100	69	17 (43.5)	15 (50.0)
Christensen	1987	74.5 ^†^	100	10	0	1 (14.3)
Boettcher	2013	72.5	100	294	16 (11.3)	16 (10.5)
Etafy	2017	63.8	100	62	0	2 (6.5)
Seifert *	1940	NA	NA	126	NA	3 (2)
Paquin *	1981	NA	NA	50	NA	6 (12)
Glahn *	1984	62	NA	300	NA	48 (16)
Circulation collapse						
Creevy	1932	67.2	100	69	0	0
Lapides	1965	NA	NA	40	NA	0
Taylor *	1966	NA	NA	18	NA	0
Glahn *	1984	62	NA	300	NA	0
Christensen	1987	74.5 ^†^	100	10	0	0
Boettcher	2013	72.5	100	294	0	0
Etafy	2017	63.8	100	62	0	0

* The data were extracted from the systemic review in 1997 [6]; ^†^ age reported as median. NA, not available.

**Table 6:** Presenting certainty of evidence (CoE) by “Grading of Recommendations Assessment, Development and Evaluation” (GRADE) methodology to assess the quality of evidence with individual endpoint.

No of Trials (No of Patients)	Risk of Bias	Inconsistency	Indirectness	Imprecision	Publication Bias	Pooled RR (95% CI)	Overall Quality of Evidence
Haematuria							
4 (435)	Very serious	Not downgraded *I*^2^ = 0.0%	Not downgraded	Downgraded	Not downgraded Begg’s test *p* = 0.497	0.91 (0.62–1.35)	⊕⊖⊖⊖ VERY LOW

CI, confidence interval; RR, relative risk; GRADE, working group grades of evidence; High certainty, we are very confident that the true effect lies close to that of the estimate of the effect; Moderate certainty, we are moderately confident in the effect estimate—the true effect is likely to be close to the estimate of the effect, but there is a possibility that it is substantially different; Low certainty, our confidence in the effect estimate is limited—the true effect may be substantially different from the estimate of the effect; Very low certainty, we have very little confidence in the effect estimate—the true effect is likely to be substantially different from the estimate of effect.

## Data Availability

The datasets used and analyzed during the current study are available from the corresponding author upon reasonable request.
